# Minerals and fatty acid profile of small indigenous fish species from homestead ponds within a Sub-tropical coastal region

**DOI:** 10.1016/j.heliyon.2024.e24445

**Published:** 2024-01-10

**Authors:** M. Belal Hossain, Rafikul Islam, Md Kamal Hossain, Afroza Parvin, Badhan Saha, As-Ad Ujjaman Nur, Md Monirul Islam, Bilal Ahamad Paray, Takaomi Arai

**Affiliations:** a School of Engineering and Built Environment, Griffith University, Nathan, QLD, Australia; b Department of Fisheries and Marine Science, Noakhali Science and Technology University, Noakhali, Bangladesh; c Laboratory of Soil, Water and Environment, Bangladesh Council of Scientific & Industrial Research, Dhaka, Bangladesh; d Nutrition Unit, Bangladesh Agricultural Research Council, Dhaka, Bangladesh; e Department of Zoology, College of Science, King Saud University, P.O. Box 2455, Riyadh 11451, Saudi Arabia; f Environmental and Life Sciences Programme, Faculty of Science, Universiti Brunei Darussalam, Bandar Seri Begawan BE 1410, Brunei Darussalam

**Keywords:** SIS, Minerals, Fatty acids, Homestead pond, Tropical coastal region, Bangladesh

## Abstract

Malnutrition has emerged as a noticeable obstruction to the socio-economic advancement of rural areas along the coastal regions of Bangladesh. Small indigenous fish species (SIS) have the potential to alleviate the malnutrition issue because of having higher nutritional compositions. However, prior research has overlooked the detailed nutritional value of SIS originating from coastal regions. Consequently, the current investigation sought to analyze the mineral and fatty acid composition of twelve SIS obtained from coastal homestead ponds. The findings indicated that the mineral composition in SIS exhibited the following descending order: calcium (Ca) > phosphorus (P) > potassium (K) > magnesium (Mg) > iron (Fe) > zinc (Zn). Furthermore, when considering the overall mineral content in SIS, it ranked in the following decreasing order: *A. testudineus*, *M. tengara*, *C. punctatus*, *N. nandus*, *P. sophore*, *C. fasciatus*, *A. mola*, *C. batrachus*, *H. fossilis*, *P. sarana*, *M. aculeatus*, and *O. pabda*. The analysis of the fatty acid profile further revealed that SIS is a rich source of palmitic acid, linoleic acid, oleic acid, stearic acid, myristic acid, palmitoleic acid, and linolenic acid. The saturated fatty acid content of the SIS varied between 42.66 % and 63.37 %, and the highest content was found in *A. mola* whereas the lowest was in *A. testudineus*. On the other hand, the total monounsaturated fatty acid content of the SIS ranged from 26.49 % (*A. mola*) to 46.12 % (*P. sarana*), and the total PUFAs contents among the fish species ranged from 5.7 % (A. mola) to 16.54 % (*H. fossilis*). Therefore, SIS could be a key source of minerals and essential fatty acids for human well-being. This can help fulfill nutrient requirements and reduce malnutrition among coastal populations. It can be said that, if these fishes are introduced in the culture systems, it will be used for consumption as well as support the livelihood of coastal people.

## 1 Introduction

Fish serves as a rich reservoir of essential nutrients crucial for enhancing the dietary needs of infants, children, and adults. In the context of Bangladesh, nearly 60 % of animal protein consumption is derived from fish, with each individual averaging an intake of 18.1 kg of fish annually [1]. Bangladesh has gained a significant position among the foremost global contributors to fish production, achieved a remarkable total fish yield of 47.59 lakh MT during the period 2021-22 [2]. Renowned as a key player in inland fishing on the global stage, Bangladesh boasts a rich aquatic landscape, harboring over 150 species of small fish out of a total of 251 distinct inland fish species [3]. As major nutrients, SIS contains protein, fat, and moisture, while carbohydrates, minerals, and vitamins are also present as minor components. It is well-known that it is rapidly digestible and includes high levels of vitamins A and D. These nutrients are essential for the development of skin, teeth, bones, and eyes. Minerals, which are mainly reserved in fish bones, are also abundant in SIS [4]. As a result, SIS is crucial in supplying rural marginal households with their sole source of animal protein.

Approximately 4.27 million rural households in Bangladesh have small ponds in their homestead areas, covering a total area of 266,259 ha [5,6]. These ponds play a significant and critical role in providing household food security and nutrition supply [7]. The majority of homestead ponds are dedicated to either extensive or improved extensive polyculture, primarily featuring rapidly growing fish species like Tilapia and Indian major carps. These ponds contribute between 25 % and 50 % of the overall fish consumption [5]. SIS are abundantly found in these culture homestead ponds, and are highly consumed in the rural Bangladeshi diet [8]. Recently, Bangladesh has been successful in reducing the total number of childhood and nutrition-related mortalities, despite various complexities. However, the maternal health status is not improving at the same pace [9]. Among pond fish farmers along the Noakhali Coast, there was a recorded prevalence of 18.8 % for malnutrition and 57.5 % for mild anemia, underscoring a relatively diminished state of health within this group [10].

SIS in homestead ponds have a short life cycle and can survive in all types of inland water reservoirs. Because of the reduction in ponds and overstocking of carp fish, SIS are under threat of extinction. These were abundantly found in ponds, beels, canals, and rivers in the past. Both natural events and human-induced disasters have contributed to the degradation of aquatic ecosystems in Bangladesh, leading to the loss of conducive environments for SIS. As a consequence, SIS are facing severe threats, and are at a critical risk of extinction [11]. In earlier times, SIS were considered as weed fish and were removed from the culture ponds using chemicals and pesticides (rotenone, benzene hexachloride, saponins, calcium cyanamide, sodium cyanide, chlorine, calcium hypochlorite, sodium pentachlorophenate, and lime etc.). Recently, this perception has changed, and SIS is now recognized as a crucial source of essential nutrients that can play a substantial role in reducing malnutrition in Bangladesh [[12], [13], [14]]. Therefore, the conservation of SIS has become a major concern among researchers in recent times, with a focus on the appropriate management of water bodies in Bangladesh. Studies have evaluated proximate composition, including ash, protein, fat, and other components like fiber and total carbohydrate in these fish [15,16]. However, the fatty acid profiles of these species are also significant, as they provide essential fatty acids like omega-6 and omega-3, which are important for various physiological functions. Moreover, fish from coastal areas can have higher concentrations of essential minerals (calcium, phosphorus, potassium, sodium, chloride and magnesium etc.), vitamins, and nutrients due to their aquatic habitat. The nutrient-rich profile of these small indigenous fish species underscores their role in sustaining healthy diets and providing alternatives to terrestrial animal-sourced foods, promoting overall well-being in the coastal area. Providing detailed factual information on nutritional values is essential for promoting this SIS to homestead pond fish farmers and the general public. Nevertheless, the nutritional composition (minerals and fatty acids) of SIS of coastal homestead pond has been less explored from homestead ponds of Noakhali Coast, Bangladesh [17]. Therefore, the present study aimed to determine the mineral and fatty acid (FA) compositions of SIS selected from the homestead ponds, and compare the variations of composition among the these SIS.

## 2 Materials and methods

### 2.1 Study area

Noakhali is a central coastal district of Bangladesh, situated between latitudes 22°07′ to 23°08′ N and longitudes 90°53′ to 91°27′ E. It is located along the watercourse near the estuary of the Meghna River and experiences a tropical climate. It has a notable precipitation rate throughout the year and a short dry season. The mean annual temperature in Noakhali is 25.2 °C, and the average annual rainfall is 3302 mm [18]. The rainfall reaches its peak in July. In contrast, January is the driest month with 8 mm of mean precipitation, and this type of climate is classified as Aw according to the Köppen–Geiger classification [19].

### 2.2 Sampling protocol

**Fig. 1:**
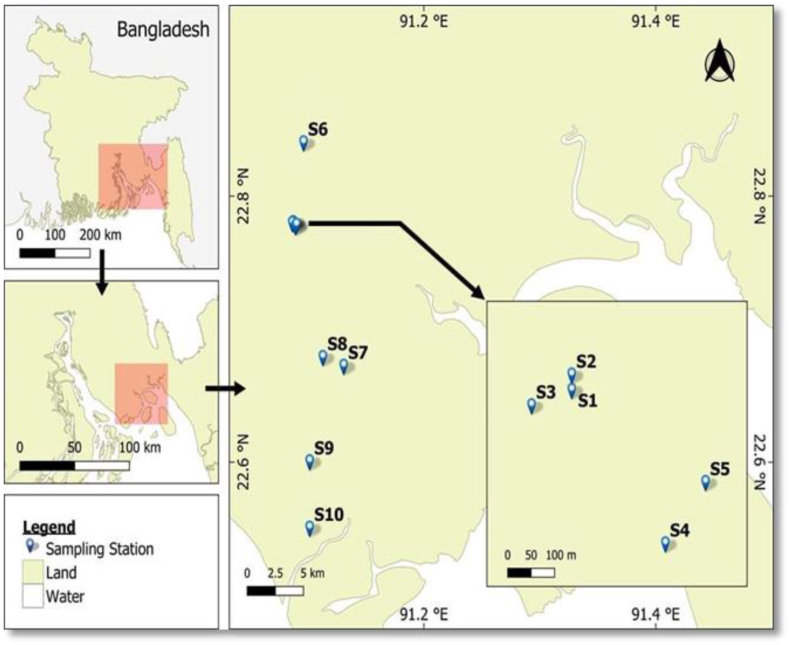
Sampling locations of homestead ponds of the Noakhali coast, Bangladesh.

A total of 10 (ten) homestead ponds (S1–S10) were chosen from the study area, which included three Upazilas (Sadar, Subarna Char, and Hatia) of Noakhali (Fig. 1). These homestead ponds, ranging from 122 to 404 m^2^ in size, are typically seasonal. They hold water for 5–6 months, starting in July, and are used for fish cultivation with extensive management. A total of 12 SIS samples (Table 1) were collected from various homestead ponds using a cast net and push net between September 2020 and October 2020. The samples were transferred to the BCSIR lab in Dhaka immediately after collection. They were transported using an insulated ice box with ice chips and stored at (−4 °C).

**Table 1:** Fish samples used for analysis of mineral and FA.

No.	Local Name	Scientific name	Length (cm)	Weight (g/fish)	Habitat
1	Khalisha	*Colisa fasciatus*	9	15	Beel, baor, pond, river
2	Mola	*Amblypharyngodon mola*	8	9	Ponds, canals, beels, and paddy fields
3	Punti	*Puntius sophore*	9.5	16	Beel, baor, pond, river
4	Sharpunti	*Puntius sarana*	24	84	Rivers, lakes, beels
5	Tarabaim	*Macrognathus aculeatus*	18	15	Ponds
6	Tengra	*Mystus tengara*	11	14	Ponds
7	Koi	*Anabas testudineus*	15	57	Ponds
8	Taki	*Channa punctatus*	18.5	88	Beel, Baor, River, pond
9	Shing	*Heteropneustes fossilis*	15.5	73	Pond, ditches, swamps and marshes
10	Magur	*Clarias batrachus*	25	113	Found in all types of freshwater bodies
11	Meni	*Nandus nandus*	13.5	46	Ponds, ditches, canals, swamps, marshes, waterlogged areas and rivers
12	Pabda	*Ompok pabda*	17	39	Canal, Beel, pond, Haor, Baor, River

### 2.3 Preparation of sample

The fins, scales (in case of scaled fish), gills, viscera of the SIS were meticulously removed and washed with tap water to eliminate blood, impurities, and slime. Afterward, the samples were subjected to conventional drying methods and subsequently ground into a fine powder using a blender (SRGR-SINGER-ELITE-RED, 650W Motor Power, China). 1 g of the powdered sample was then placed in a 50 ml beaker and combined with 10 ml of concentrated HNO_3_ (analytical grade, 69 % w/w). This mixture was then positioned on a magnetic hot plate stirrer, with the initial temperature set at 40 °C for 1 h to mitigate excessive reactions. The temperature was then elevated to 140 °C and upheld for a duration of 3 h. After that, the mixture was allowed to cool at room temperature, and 5 ml of H_2_O_2_ was introduced to the mixture. The solution was again heated on a hot plate until it turned transparent. Upon completion of the digestion process, the samples were filtered into a 50 ml flask using Whatman No. 44 filter paper for further analysis into Atomic Absorption Spectrophotometer (AAS).

### 2.4 Analytical method for minerals

The samples underwent analysis utilizing the Atomic Absorption Spectrophotometer (AAS, Shimadzu model AA-7000, manufactured in Tokyo, Japan), following established analytical protocols [20]. The AAS instrument was configured with the appropriate flame settings, and the measurement conditions were optimized for the analyses. Subsequently, distilled deionized water (DDW) blanks, standards, and samples were introduced into the AAS flame. The calibration curves, generated by correlating concentration with absorbance, underwent statistical assessment using the least square method to establish linear fitting. Initial blank readings were recorded, and adjustments were applied during the subsequent calculations as needed.

### 2.5 Quality assurance

Two blank samples with reagent only were conducted in each batch. Twelve samples from each individual were analyzed. Distilled deionized water (DDW) was used for sensing and preparing reagents. Additionally, analytical-grade (69 % w/w) reagents were used in this study.

### 2.6 Formula for minerals analysis

Potassium (K), calcium (Ca), iron (Fe), magnesium (Mg), zinc (Zn) and phosphorus (P) were determined using the following formula:

$$Minerals\;Concentration=\frac{\left(\text{Reading}-\text{Blank}\;\text{reading}\right)\times \text{Final}\;\text{volume}\;\text{of}\;\text{sample}\;\text{in}\;\text{ml}}{\text{Weihgt}\;\text{of}\;\text{fish}\;\text{sample}\;\text{taken}\;\text{in}\;g}$$

### 2.7 Analytical methods for fatty acid

Fat and fatty acids (FA) were isolated from the samples through a hydrolytic process. Fat was extracted using ether and subsequently transformed into Fatty Acid Methyl Ester (FAME). Quantitative assessment of FAMEs was conducted using gas chromatography (GC, Shimadzu GC-14B, Japan). The fatty acid (FA) composition of the chosen species was determined employing gas chromatography. The FA composition was derived from lipids via saponification, facilitated by a mixture of NaOH dissolved in methanol and water [21]. The proportional concentration of fatty acids (FA) was deduced from oil samples, where their corresponding methyl esters were analyzed [22], with a slight adaptation. After adding 3 ml of 0.5 M sodium methoxide, a 15 ml test tube held 5–7 droplets of oil. In a boiling water bath, the mixture was heated and stirred for 15 min. After cooling to room temperature, 1 ml of petroleum ether (boiling point 40–60 °C) was introduced, followed by 10 ml of deionized water. Subsequent thorough mixing was followed by 5 min settling period, after which top layer of the petroleum ether-methyl ester mixture was put into a sealed vial. In parallel, 200 mg of diverse fatty acid (FA) standards in their respective methyl ester forms were separately diluted in 10 ml of petroleum ether (boiling point 40–60 °C). Aliquots of 1 μl of FAME were inserted, and the peaks of FAs were noted for their corresponding retaining times and areas, utilizing the data processing unit of a gas chromatography. The apparatus featured a fused silica capillary column and a flame ionization detector (FAMEWAX, Crossbond® polyethylene glycol, Restek, Pennsylvania, USA). A pristine injection technique employed nitrogen as the carrier gas at a consistent flow rate of 20 ml per minute. The injector temperature was maintained at 250 °C, while the initial oven temperature was set at 150 °C for a 5 min hold. Subsequently, the temperature escalated at a rate of 8 °C per minute until reaching 190 °C, then gradually increased to 200 °C at a rate of 2 °C per minute, maintaining this temperature for 10 min. Fatty acids (FAs) were subsequently identified through their respective FAME standards and expressed as relative percentages computed by the automated GC software (Class GC-10, version-2.00).

### 2.8 Data analysis

The mean concentration of minerals and FAs among the species were analyzed by using the PAST (PAleontological Statistics; Version 4.03) software. Additionally, homogeneity of variances and data transformations were carried out using Levene's test and the square root or logarithm when needed. The study area map was conducted by ArcGIS (version 10.7) and Origin-Pro software was used to illustrate rest of the figures.

## 3 Results and discussion

### 3.1 Micronutrients in SIS

**Fig. 2:**
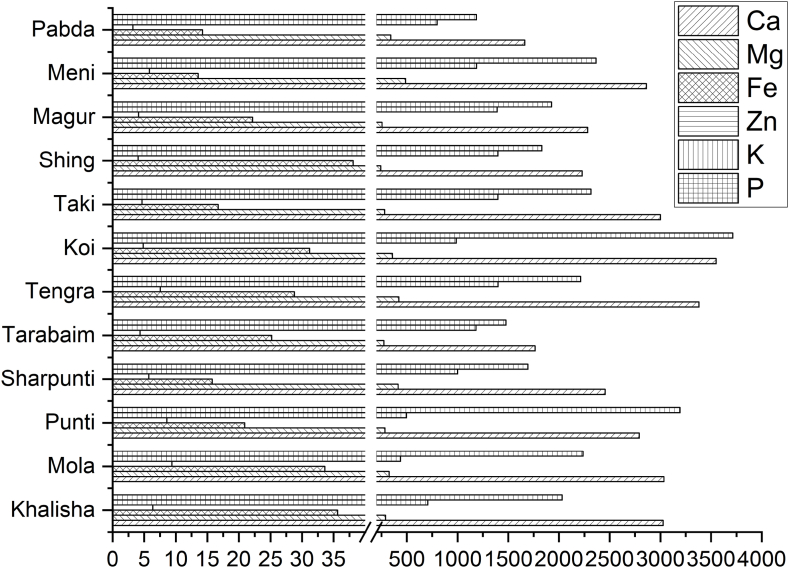
Minerals contents of some SIS (Nutrient content in mg/g of dry sample).

The composition of Ca, Mg, Fe, Zn, K, and P for all species is shown in Table 2. The micronutrients in the SIS followed the decreasing order of Ca (26.71 ± 5.98 mg/g), P (21.83 ± 6.98 mg/g), K (10.33 ± 3.54 mg/g), Mg (3.32 ± 0.76 mg/g), Fe (0.25 ± 0.09 mg/g), and Zn (0.06 ± 0.02 mg/g). The mineral contents in the selected SIS followed the decreasing order of *A. testudineus*, *M. tengara*, *C. punctatus*, *N. nandus*, *P. sophore*, *C. fasciatus*, *A. mola*, *C. batrachus*, *H. fossilis*, *P. sarana*, *M. aculeatus* and *O. pabda* (Fig. 2).

**Table 2:** Minerals contents of some SIS (Nutrient content in mg/g of dry sample).

Species	Ca	Mg	Fe	Zn	K	P
*C. fasciatus*	30.27 ± 0.28	2.91 ± 0.05	0.36 ± 0.03	0.06 ± 0.004	7.06 ± 0.07	20.31 ± 0.06
*A. mola*	30.36 ± 0.16	3.26 ± 0.07	0.34 ± 0.02	0.09 ± 0.003	4.39 ± 0.06	22.38 ± 0.08
*P. sophore*	27.92 ± 0.27	2.85 ± 0.06	0.21 ± 0.02	0.09 ± 0.005	4.95 ± 0.05	31.94 ± 0.13
*P. sarana*	24.56 ± 0.22	4.15 ± 0.09	0.16 ± 0.01	0.06 ± 0.003	10.00 ± 0.09	16.95 ± 0.07
*M. aculeatus*	17.64 ± 0.24	2.75 ± 0.08	0.25 ± 0.01	0.04 ± 0.003	11.82 ± 0.06	14.77 ± 0.11
*M. tengara*	33.82 ± 0.30	4.22 ± 0.03	0.29 ± 0.02	0.08 ± 0.004	14.00 ± 0.07	22.14 ± 0.05
*A. testudineus*	35.51 ± 0.23	3.58 ± 0.05	0.31 ± 0.02	0.05 ± 0.004	9.86 ± 0.07	37.13 ± 0.16
*C. punctatus*	30.01 ± 0.17	2.82 ± 0.05	0.17 ± 0.02	0.05 ± 0.004	13.98 ± 0.07	23.16 ± 0.10
*H. fossilis*	22.29 ± 0.26	2.42 ± 0.04	0.38 ± 0.02	0.04 ± 0.002	13.97 ± 0.06	18.32 ± 0.09
*C. batrachus*	22.84 ± 0.15	2.56 ± 0.05	0.22 ± 0.01	0.04 ± 0.002	13.91 ± 0.05	19.27 ± 0.08
*N. nandus*	28.62 ± 0.18	4.88 ± 0.06	0.14 ± 0.01	0.06 ± 0.004	11.88 ± 0.06	23.66 ± 0.13
*O. pabda*	16.63 ± 0.17	3.42 ± 0.04	0.14 ± 0.01	0.03 ± 0.002	7.98 ± 0.07	11.87 ± 0.08

#### 3.1.1 Calcium (Ca) content

Ca content ranged from 16.45 to 35.78 mg/g, with a mean content of 26.71 ± 5.98 mg/g. The maximum load of Ca (35.51 ± 0.23 mg/g) was found in *A. testudineus*, while the minimum load was found in *O. pabda* (16.63 ± 0.17 mg/g). The findings showed a wider range in calcium content compared to the values reported in the global FAO/INFOODS database [23]. This might happen as they analyzed fish fillets without bones and fish heads.

The higher content of calcium (Ca) in SIS was recorded due to the presence of fish bones. Bones are the primary source of calcium in fish [24]. Dairy products like butter, milk, and cheese are the main source of dietary calcium in industrialized nations, but are uncommon in Bangladesh [25]. Previous research conducted in the Kishoreganj district of Bangladesh revealed that the average daily fish consumption rate was 65 g/person, which can meet 31 % of the average daily calcium requirement [26]. Therefore, SIS could be an important source of highly bioavailable dietary calcium in Bangladesh [27,28].

#### 3.1.2 Magnesium (Mg) content

The Mg load in SIS from homestead ponds ranged from 4.94 to 2.38 mg/g, with an average of 3.32 ± 0.76 mg/g. These values were similar to those found in previous studies [23]. The Mg content in SIS followed a decreasing order among the following fish species: *N. nandus, M. tengara, P. sarana, A. testudineus, O. pabda, A. mola, C. fasciatus, P. sophore, C. punctatus, M. aculeatus, C. batrachus,* and *H. fossilis*. The present study obtained a higher concentration of Mg compared to other studies, indicating that the selected SIS is a very good source of Mg [29]. The daily recommended intake of magnesium (Mg) for adults is 220–260 mg [30], and this can be met by consuming SIS daily.

#### 3.1.3 Iron (Fe) content

Fe varied from 0.13 to 0.39 mg/g in the studied SIS, with an average of 0.25 ± 0.09 mg Fe/g. The highest content of Fe was recorded as 0.38 ± 0.02 mg/g in *H. fossilis*, while the lowest was 0.14 ± 0.01 mg/g in *N. nandus* and *O. pabda*. In this study, the Fe content in *P. sophore* was recorded as 0.21 ± 0.02 mg/g, while the previous study [29] recorded 0.12 mg Fe/g. Further, 0.14 mg Fe/g was found in *A. mola* [31] which is lower than the current findings of 0.34 ± 0.02 mg/g. The findings of this study were higher than those obtained by Bogard et al. [24] and Gopakumar [32]. This is because they only analyzed the raw edible parts, whereas the present study analyzed the whole body. Fe is highly concentrated in the fish head and viscera. However, the recommended intake of Fe for females aged 15–50 years is 24 mg/day. Previous studies have shown that daily consumption of *P. sophore* can help meet 38 % of the iron needs of lactating women [29]. Another study revealed that consuming SIS with boiled rice may supply, on average, 45 % of the daily Fe requirement in infant women and 42 % in children [[26], [33]]. Hence, the SIS can provide daily iron requirements and also ensure a sufficient supply of Fe for each day. There may be changes in environmental factors, fish diets, species, and water quality that account for the variance in Fe levels among species.

#### 3.1.4 Potassium (K) content

The potassium (K) content varied significantly, ranging from 4.33 to 14.06 mg/g, with a mean of 10.31 ± 3.54 mg/g. The K content followed a decreasing order as follows*: M. tengara*, *C. punctatus*, *H. fossilis*, *C. batrachus*, *N. nandus*, *M. aculeatus*, *P. sarana*, *A. testudineus*, *O. pabda*, *C. fasciatus*, *P. sophore*, and *A. mola*. This finding was also higher than that of Bogard et al. [24], Mohanty et al. [29], and Mohanty et al. [34] given that they only examined the species' raw edible portions. In this study, the highest K concentration (14.0 ± 0.07 mg/g) was obtained from *M. tengara*. The daily recommended intake of potassium for adult males aged 25–50 years is 800 mg, which is consistent with the findings of the present study. From the study, consuming SIS will meet the daily requirement of K properly, assuming that cooking will not affect the quality of the minerals [35,36].

#### 3.1.5 Phosphorus (P) content

In this study, the phosphorus (P) content in the examined SIS ranged from 11.78 to 37.31 mg/g. The concentration of P was found to be high, followed by Ca. As the samples were analyzed using the entire body, the SIS showed the highest concentration of Ca and P. For instance, the fish's bones, viscera, and scales retain over 99 % of the collected calcium and 80 % of the accumulated phosphorus, with the remaining 1 % being dispersed throughout the remainder of the body [37]. Therefore, the present findings were higher than the results of Bogard et al. [24] in *A. testudineus* (1.6 mg/g), *H. fossilis* (2.2 mg/g), *C. batrachus* (2.1 mg/g), *N. nandus* (8.1 mg/g), *O. pabda* (1.5 mg/g), and Mohanty et al. [29] in *P. sarana* (2.68 mg/g), *C. punctatus* (5.35 mg/g), *H. fossilis* (1.36 mg/g), and *C. batrachus* (1.22 mg/g). Hossain et al. [38] found that the Ca/P ratio in twenty-three small indigenous fish species (SIS) range between 0.44 and 2.00. The total amount of P in the human body is approximately 700 g, with 80 % stored in the bones, 10.9 % in the viscera, and the remaining amount in the skeletal muscle tissue [39,40]. So, SIS can play a significant role in the improvement of human health.

### 3.2 Fatty acid composition of SIS

The composition of fatty acids in the studied SIS was analyzed using gas chromatography and is presented in Table 3. The findings revealed that saturated fatty acids (SFAs) were the highest in SIS from homestead ponds, followed by monounsaturated fatty acids (MUFAs) and polyunsaturated fatty acids (PUFAs) in the species. Fifteen types of fatty acids (FAs) were identified among the species: myristic acid (C14:0), myristoleic acid (C14:1), palmitic acid (C16:0), stearic acid (C18:0), arachidic acid (C20:0), behenic acid (C22:0), lignoceric Acid (C24:0), oleic acid (C18:1), linoleic acid (C18:2), linolenic acid (C18:3), arachidonic acid (C20:4), eicosenoic acid (C20:1), eicosapentaenoic acid (EPA) (C20:5), and docosahexaenoic acid (DHA) (C22:6).

**Table 3:** Fatty acid composition of selected Small Indigenous Fish.

Fatty acid	*C. fasciatus*	*A. mola*	*P. sophore*	*P. sarana*	*M. aculeatus*	*M. tengara*	*A. testudineus*	*C. punctatus*	*H. fossilis*	*C. batrachus*	*N. nandus*	*O. pabda*
**Saturated Fatty Acid %**	**59.6951**	**63.3666**	**44.4938**	**43.6388**	**47.0367**	**49.8642**	**42.656**	**47.9053**	**47.9696**	**45.4735**	**53.8977**	**51.1809**
a) Myristic acid (C 14:0)	5.1015	9.5401	2.812	1.5586	2.3165	2.9678	1.3772	3.2635	2.9461	2.7935	4.6198	1.2789
b) Palmitic acid (C 16:0)	50.6128	52.0627	36.7169	36.3769	39.2524	42.0781	37.1274	40.1021	40.3226	38.1317	44.8383	46.2665
c) Stearic acid (C 18:0)	3.5584	1.5673	3.3353	4.27	3.2154	2.9764	3.5777	3.5033	3.0169	3.1909	3.8348	3.0023
d) Arachidic acid (C 20:0)	0.1649	0.1965	0.9493	0.6928	1.5478	1.1173	0.1574	0.4704	1.0859	1.0245	0.0931	0.3316
e) Behenic acid (C 22:0)	–	–	–	0.0866	0.5621	0.4758	0.2686	0.2201	0.4053	0.2565	0.2129	–
f) Lignoceric Acid (C 24:0)	0.2575	–	0.0575	0.6539	0.1425	0.2488	0.1477	0.3459	0.1928	0.0764	0.2988	0.3016
**Unsaturated fatty acids %**	**40.3049**	**36.6334**	**55.5062**	**56.3612**	**52.9633**	**50.1358**	**57.3439**	**52.0946**	**52.0303**	**54.5265**	**46.1022**	**50.1358**
**i)Monounsaturated fatty acids %**	**34.6018**	**26.4921**	**46.1181**	**46.1222**	**40.0382**	**41.6202**	**41.3308**	**40.4292**	**35.4909**	**41.3779**	**36.9642**	**40.3458**
a) Myristoleic acid (C 14:1)	5.1329	2.6845	2.8565	0.6448	1.9853	2.2748	–	1.7927	2.8957	3.1257	3.1381	0.1759
b) Palmitoleic acid (C 16:1)	4.4635	1.0533	3.3353	5.2156	2.539	2.8502	0.3592	2.2944	0.5183	2.9765	1.995	0.2564
c) Oleic acid (C 18:1)	24.8893	22.7543	38.9012	39.5138	35.4265	36.4952	40.7929	36.3421	31.8508	35.0961	31.7403	39.7285
d) Eicosenoic acid (C 20:1)	0.1161	–	1.0251	0.748	0.0875	–	0.1787	–	0.2261	0.1796	0.0908	0.185
**ii)Polyunsaturated fatty acids %**	**5.7031**	**10.1414**	**9.3881**	**10.2392**	**12.9251**	**8.5158**	**16.0131**	**11.6654**	**16.5394**	**13.1486**	**9.138**	**8.4735**
a) Linoleic acid (C 18:2)	3.7764	3.7554	8.4614	9.7211	11.1872	6.0303	15.1436	8.7753	8.0988	6.3432	6.7438	7.6
b) Linolenic acid (C 18:3)	1.2166	5.2155	0.6421	0.1475	1.6109	1.8724	0.5059	1.1508	5.3832	3.3364	0.8192	0.0758
c) Arachidonic acid (C 20:4)	0.2505	0.3359	0.058	0.2403	0.0608	0.1645	–	0.8039	1.8838	2.357	0.7909	–
d) Eicosapentaenoic acid (EPA) (C20:5)	0.2175	–	0.1885	0.0434	0.0093	–	–	0.1428	0.2009	0.5668	0.0748	0.191
e) Docosahexaenoic acid (DPA) (C 22:6)	0.2415	0.3251	0.0381	0.0869	0.0569	0.4486	0.3636	0.7926	0.9627	0.5451	0.7093	0.6067

#### 3.2.1 Saturated fatty acid (SFA)

The total saturated fatty acid (SFA) content of the fish species ranged from 42.66 % to 63.37 %. The Mola (*A. mola*) had the highest SFA content, while the Koi (*A. testudineus*) had the lowest. The percentage of SFAs followed a decreasing order: *A. mola* (63.37 %), *C. fasciatus* (59.7 %), *N. nandus* (53.9 %), *O. pabda* (51.18 %), *M. tengara* (49.86 %), *H. fossilis* (47.97 %), *C. punctatus* (47.91 %), *M. aculeatus* (47.04 %), *C. batrachus* (45.47 %), *P. sophore* (44.49 %), *P. sarana* (43.64 %), and *A. testudineus* (42.66 %). The main saturated fatty acids (SFAs) found in the tested SIS were myristic acid (C14:0), palmitic acid (C16:0), and stearic acid (C18:0). The highest levels of myristic acid (9.54 %), palmitic acid (52.06 %), and stearic acid (4.27 %) were found in *A. mola* and *P. sarana*, respectively. The lowest levels of myristic acid (1.28 %), palmitic acid (36.38 %), and stearic acid (1.57 %) were found in *O. pabda, P. sarana*, and *A. mola*, respectively. The SFAs contents in *A. mola* and *P. sophore* coincide with the findings of Dey et al. [41]. Palmitic acid (C16:0) was the main fatty acid, accounting for 52–85 % of the total SFA. The highest palmitic acid content was 52.06 % in *A. mola* while the lowest value was 36.38 % in *P. sarana*. Mustafa et al. [42] found C16:1 ω7 and C18:1 ω-9 SFAs which are more or less similar than our present findings. Zuraini et al. [43] found comparable values of palmitic acid (25.63 ± 1.45 % to 30.39 ± 0.23 %) (0.13–0.27 g/g) for three *Channa* spp. fish from Malaysia.

#### 3.2.2 Monounsaturated fatty acids (MUFAs)

The total MUFA content of the experimented SIS ranged from 26.49 % to 46.12 %, with the highest content found in *A. mola* and the lowest in *P. sarana*. The predominant monounsaturated fatty acids (MUFAs) in the studied SIS were oleic acid (C18:1) and a minor percentage of myristoleic acid (C14:1), palmitoleic acid (C16:1) and eicosenoic acid (C20:1). The highest level of oleic acid (40.79 %) was found in Koi (*A. testudineus*), while the lowest level (22.75 %) was found in Mola (*A. mola*), respectively. But eicosenoic acid (C20:1) was not detected in *A. mola*, *M. tengara*, and *C. punctatus*, respectively. This is similar to the study carried out by Dey et al. [41], where they revealed a MUFA content (0.25–0.48 g/g) in River Indus fishes. The level of MUFAs was 37.12 ± 0.01 % in *P. sophore*, as studied by Mohanty et al. [29], which is lower than the level found in the present study. The highest level of predominant oleic acid (40.79 %) was found in *A. testudineus*, while the lowest level (22.75 %) was found in *A. mola*, respectively.

#### 3.2.3 Polyunsaturated fatty acids (PUFAs)

The total PUFA content of the fish species ranged from 5.7 % to 16.54 %. The highest PUFA content was found in Mola (*A. mola*), while the lowest was found in Shing (*H. fossilis*). The predominant polyunsaturated fatty acids (PUFAs) in the studied fishes were linoleic acid (C18:2) and linolenic acid (C18:3). Other minor contents of PUFAs included arachidonic acid (C20:4), eicosapentaenoic acid (EPA) (C20:5), and docosahexaenoic acid (DHA) (C22:6). The highest level of linoleic acid (15.14 %) was found in *A. testudineus*, while the lowest level (3.78 %) was found in *A. mola*, respectively. Eicosapentaenoic acid (EPA) (C20:5) was not detected in *M. tengara* and *C. punctatus*, respectively. Sargent et al. [44] reported that n-3 PUFA, primarily DHA, plays a significant role in neural cell membranes. Furthermore, it is considered an essential component for maintaining human nutrition and health. Freshwater fish have the ability to convert linolenic acid into longer n-3 PUFA through chain elongation and desaturation. The concentration of arachidonic acid (AA) (C20:4 n-6) varies from 0.0580 % to 2.3570 % among different fish species. Bowman and Rand [45] reported that AA is a precursor for prostaglandins and thromboxanes, which influence blood clot formation and their attachment to the endothelial tissue during wound healing. Njoroge et al. [46] recently showed that EPA and DHA can reverse fatty acid abnormalities associated with cystic fibrosis. McAlonan et al. [47] stated that it has already examined the effectiveness of regularly taking PUFAs in reducing attention deficit hyperactivity disorder (ADHD), a neurobehavioral disorder. Besides, omega-3 and omega-6 PUFAs are considered important. As these cannot be produced in the human body, people must obtain them from their diet.

Diverse and significant fatty acids were found in fish. The variation in crude fat content among fish species may be influenced by factors such as water temperature, salinity, life stage, and diet [48]. The FA profile of the fishes revealed that the majority of them have a higher concentration of unsaturated fatty acids (UFA) compared to saturated fatty acids (SFA). The health friendly PUFA, DHA, and EPA content varied notably between the species. As PUFAs have numerous beneficial effects on human health, SIS may have a positive impact on improving the nutritional health status of impoverished individuals suffering from malnutrition in the coastal region.

## 4 Conclusion

The goal is to ensure both nutritional and economic benefits. The data presented here reveals that SIS has the potential to significantly increase the ratio of mineral and fatty acid intake for the vulnerable coastal community. The results revealed that the mineral contents in 12 selected SIS followed a decreasing order of Ca (26.71 ± 5.98 mg/g), P (21.83 ± 6.98 mg/g), K (10.33 ± 3.54 mg/g), Mg (3.32 ± 0.76 mg/g), Fe (0.25 ± 0.09 mg/g), and Zn (0.06 ± 0.02 mg/g). The analysis of the fatty acid profile further revealed that SIS is a rich source of palmitic acid, stearic acid, oleic acid, myristic acid, linoleic acid, palmitoleic acid, and linolenic acid. The total content of saturated fatty acids (SFA) in SIS ranged from 42.66 % (*A. testudineus*) to 63.37 % (*A. mola*). On the other hand, the overall MUFA content in the fish species varied from 26.49 % (*A. mola*) to 46.12 % (*P. sarana*). Furthermore, the overall content of PUFAs varied among the fish species, ranging from 5.7 % (*A. mola*) to 16.54 % (*H. fossilis*). This suggests that SIS could serve as a significant source of essential minerals and fatty acids for human consumption. By incorporating SIS into their diet, coastal communities can meet their nutritional needs and reduce the prevalence of malnutrition. Further study has been suggested to improve culturing methods and protect endangered fish species. This will enable nutritionists to easily formulate the dietary requirements of fish for daily meals and provide guidelines for the general population to maintain a nutritious diet plan for optimal health. This is particularly important for pregnant and lactating women, as well as children.

## Funding

This study was supported by Food Based Project, PIU, NATP-2, 10.13039/100020982BARC (Grant ID-011), Bangladesh. This study was partially supported by the Universiti Brunei Darussalam under the Faculty/Institute/Centre Research Grant (No. UBD/RSCH/1.4/FICBF(b)/2023/057). In addition, this study was supported by Researchers Supporting Project Number (RSP2024R144), 10.13039/501100002383King Saud University, Riyadh, Saudi Arabia.

## Additional information

No additional information is available for this paper.

## CRediT authorship contribution statement

**M. Belal Hossain:** Writing – review & editing, Resources, Project administration, Methodology, Funding acquisition, Conceptualization. **Rafikul Islam:** Methodology, Investigation, Formal analysis. **Md Kamal Hossain:** Methodology, Formal analysis, Data curation. **Afroza Parvin:** Resources, Methodology, Investigation, Formal analysis. **Badhan Saha:** Methodology, Investigation, Funding acquisition, Formal analysis, Data curation. **As-Ad Ujjaman Nur:** Software, Resources, Project administration, Formal analysis, Conceptualization. **Md Monirul Islam:** Investigation, Funding acquisition, Conceptualization. **Bilal Ahamad Paray:** Writing – review & editing, Validation, Project administration, Funding acquisition. **Takaomi Arai:** Writing – review & editing, Visualization, Resources.

## Declaration of competing interest

The authors declare the following financial interests/personal relationships which may be considered as potential competing interests: Bilal Ahamad Paray reports financial support was provided by 10.13039/501100002383King Saud University.
